# Development and delivery of an SMS-based remote monitoring and support service: ‘Simple Telehealth’

**Published:** 2012-06-15

**Authors:** David Barrett, Phil O’Connell, Jeremy Wyatt

**Affiliations:** University of Hull, UK; NHS Stoke-on-Trent, UK; Institute of Digital Healthcare, University of Warwick, UK

**Keywords:** telemonitoring, mHealth, SMS, mobile

## Abstract

**Introduction:**

Healthcare delivery faces unprecedented pressure due to a combination of an aging population, increasing prevalence of long-term conditions (LTCs), and public spending austerity. To mitigate these pressures, technology is increasingly being utilised to promote self-care and enable remote, less intensive support from healthcare professionals. ‘Telehealth’ is a term used to describe the use of technology to remotely support healthcare and promote well-being. This paper describes the development and impact of one such mobile phone-based application—‘Simple Telehealth’.

**Objective:**

The objective of the Simple Telehealth project was to develop an innovative, low-cost system for remotely supporting healthcare delivery and promoting well-being.

**Methods:**

Simple Telehealth utilises SMS messaging to facilitate communication between patients, health and social care practitioners, and decision support software. Patients use a mobile phone to send and receive messages to and from the service support software (‘Florence’), and are provided with point-of-care testing devices for physiological measurement (such as electronic sphygmomanometers or thermometers). Patients are advised to take regular measurements and text results to Florence in a standard format (e.g. BP 100 60 temperature 36.7). Patients are registered on the Florence server by clinicians using a simple web interface. Florence prompts patients to take readings, receives incoming texts, compares measurements to pre-set parameters and provides automated SMS-based feedback to patients. Florence also sends reminder texts according to a practitioner-defined regime, and enables direct SMS-messaging between practitioners and patients. In addition, Florence acts as a shared repository of patient data that can be viewed by health and social care practitioners to support collaborative working.

**Results to date:**

Since its launch in 2010, over 1000 patients have been registered on Simple Telehealth. The service has been used by 300 clinicians in 45 clinical teams. User acceptability and satisfaction is high, and the system anecdotally provides benefits to healthcare practitioners. Simple Telehealth facilitates large-scale, low-cost telemonitoring programmes utilising existing lifestyle technology rather than bespoke, high-cost systems. It is anticipated that the telemonitoring functionality will bring quality of life and clinical benefits similar to those reported in recent Cochrane Reviews. Giving patients a greater understanding of their health status should also enhance self-care ability and—where appropriate—underpin behaviour change. We believe that Simple Telehealth can reduce the frequency of community visits, bringing immediate productivity savings in consumables and travel costs. In addition, the local health community benefits through reduced tariff costs for A&E attendances and hospital admissions. Micro-cases that simulate the economic benefits of Simple Telehealth have identified potential return on investment ratios of approximately 10:1. Building on initial successes, next-stage developments include enhanced links with social care alarm response services, Bluetooth-enabled physiological measurement and voice recognition functionality for users in whom SMS text entry proves difficult.

**Conclusions:**

Simple Telehealth provides a user-friendly and robust platform for the enhanced monitoring and support of patients with LTCs. Despite—or possibly because of—using basic technologies, the system has proven popular with healthcare professionals and patients alike, and has the potential to deliver substantial efficiency savings to healthcare *organisations*.

